# Mandibular Carnassial Tooth Malformations in 6 Dogs—Micro-Computed Tomography and Histology Findings

**DOI:** 10.3389/fvets.2019.00464

**Published:** 2019-12-17

**Authors:** Kevin K. Ng, Stacy Rine, Eunju Choi, Nadine Fiani, Ian Porter, Lisa Fink, Santiago Peralta

**Affiliations:** ^1^Department of Clinical Sciences, College of Veterinary Medicine, Cornell University, Ithaca, NY, United States; ^2^Department of Biomedical Sciences, College of Veterinary Medicine, Cornell University, Ithaca, NY, United States; ^3^Department of Pathology, Microbiology and Immunology, School of Veterinary Medicine, University of California, Davis, Davis, CA, United States; ^4^Arizona Veterinary Dental Specialists, Scottsdale, AZ, United States

**Keywords:** dens invaginatus, micro-computed tomography, dental malformation, enamel pearl, histopathology

## Abstract

**Objective:** To document the clinical, radiographic, and histological characteristics of mandibular first molar teeth with developmental abnormalities previously attributed to dens invaginatus and enamel pearls in dogs.

**Materials and Methods:** Affected mandibular first molar teeth from dogs were evaluated grossly and via intraoral radiography. Endodontically and/or periodontally compromised teeth were extracted and subjected to some combination of micro-computed tomography, histopathology, and immunohistochemistry with anti-amelogenin antibody.

**Results:** Six dogs with developmental abnormalities of mandibular first molar teeth were identified, representing 11 affected teeth. The condition was bilateral in 5 dogs, while in 1 dog, only one mandibular first molar tooth was present. Patient weight ranged from 1.7 to 6 kg (median = 4.09 kg). On intraoral radiographs, root convergence or parallelism was noted in 6 of 11 teeth, and root dilaceration was noted in 3 of 11 teeth. Eight teeth required extraction due to periapical lucencies or periodontitis. On micro-CT, the abnormal teeth were characterized by the presence of abnormal, heterogenous hard tissue with beam attenuation characteristics midway between that of enamel and dentin. Enamel fissures were identified in 4 of 8 teeth, while ectopic radicular enamel was identified in 2 of 8 teeth. The abnormal tissue was traversed by channels measuring 20–40 μm in diameter. Channels communicated with the enamel fissures in 2/8 teeth, the furcation in 2/8 teeth and the pulp in 4/8 teeth. The abnormal tissue was frequently surrounded by disorganized dentin. Histologic features of enamel and dentin were absent from the abnormal tissue and immunohistochemistry to detect amelogenin in the abnormal tissue was negative in all samples.

**Conclusion:** The dental abnormalities described here correspond to a previously unrecognized developmental abnormality involving the mandibular first molar teeth in dogs. The developmental origin of the abnormal tissue could not be ascertained, and further investigations are required to determine the mode of formation, origin of the abnormal tissue, and factors associated with development. These developmental abnormalities more closely resemble molar-incisor malformation, rather than dens invaginatus or enamel pearls as described in humans. The authors propose that affected mandibular first molar teeth simply be referred to as having carnassial tooth malformations.

## Introduction

Several types of dental developmental abnormalities have been described in dogs (1, 2) and humans (3). Dental developmental abnormalities are clinically relevant because they can result in aberrant pulp chamber anatomy that predisposes affected teeth to pulp necrosis, or structural irregularities that create plaque-retentive surfaces which predispose periodontitis or caries lesion formation (3). Understanding the morphological and clinical features of developmental abnormalities of teeth is essential to better understand the possible etiological mechanisms involved, and to elucidate preventive and therapeutic solutions that could be of veterinary and comparative value.

One developmental abnormality that has been previously documented in dogs appears to preferentially affect mandibular first molar teeth (4), also known as the mandibular carnassial teeth. The lesions frequently occur bilaterally (5–11) and are characterized by certain features when they affect the mandibular first molar teeth. The most common feature appears to be abnormal radiopaque structures within the crown (7, 9–11), but furcation abnormalities (6), abnormalities in coronal enamel (5, 7–10), and root convergence (9, 10) have also been reported with varying regularity. Periapical lucencies (6–10) and periodontitis (5, 6, 8–10) are frequently evident and highlight the clinical significance of this condition.

Such abnormalities have been attributed to a form of dens invaginatus (7–10), or enamel pearl formation (1, 5). However, the apparent predilection of this condition for bilateral involvement of the mandibular first molar teeth differs from that reported in dens invaginatus (12) and enamel pearls (13) in humans. Furthermore, descriptions of these lesions have primarily relied on radiographic findings and gross appearance (1, 4–10). Advanced imaging modalities have not been utilized to investigate its morphological features and histopathological analysis has only been applied in two reports (6, 11). Therefore, it is unclear whether mandibular first molar teeth with abnormalities previously attributed to dens invaginatus or enamel pearls truly correspond to these conditions as described in humans, or if they represent a different dental anomaly.

The aims of this study were to systematically document the morphological characteristics of mandibular first molar teeth with developmental abnormalities previously attributed to dens invaginatus and enamel pearls based on gross examination, conventional dental radiography, micro-computed tomography (micro-CT), and histopathological analysis.

## Materials and Methods

### Case Selection, Tissue Collection, and Intraoral Radiography

Dogs were prospectively recruited from patients presenting to the Dentistry and Oral Surgery Service at the Cornell University Hospital for Animals between 2016 and 2018 for the treatment of oral disease. Animals in which an abnormal radiopaque structure was noted within one or both mandibular first molar teeth on routine full-mouth dental radiographs were included. Specifically, teeth were considered to have this condition if they had a radiopaque structure of dentin or higher opacity within the tooth that resulted in deviation of the pulp chamber on intraoral radiographs. This inclusion criterion was chosen as it appears to be the most common clinical or radiographic feature among mandibular first molar teeth with this abnormality. One additional dog that was presented to the Cornell University Veterinary Specialists hospital and met similar criteria, was recruited.

All dogs had a complete physical examination performed, and either point-of-care bloodwork (packed cell volume, serum total protein, blood urea nitrogen, blood glucose) or a complete blood count and serum biochemistry profile was performed prior to general anesthesia. The anesthetic protocol was determined by a board-certified veterinary anesthesiologist, or by the board-certified veterinary dentist supervising the case. Full-mouth radiographs were obtained using dedicated dental intraoral radiographic equipment and intraoral photostimulable phosphor plate (PSP) systems (CS 7600, Carestream Dental, Atlanta, GA; Scan X Duo, Air Techniques, Melville, NY) using standard projections (14). Images were evaluated using dedicated digital dental radiography software (Dental Imaging Software, Carestream Dental, Atlanta, GA; Tigerview, Televere Systems, Janesville, WI) by board-certified veterinary dentists (NF, SP, LF) and a veterinary dentistry and oral surgery resident in training (KKN). Conclusions were established by consensus.

Based on gross examination, the presence of enamel abnormalities appearing as a depression or groove, hereafter referred to as an “enamel fissure,” was recorded. The presence of root convergence or dilaceration on intraoral radiography was also recorded. Additionally, the presence of pulpal opacities in other teeth other than the mandibular first molars was recorded based on evaluation of full-mouth radiographs. Finally, the presence of periapical lucencies was recorded, and the severity of periodontitis, if present, was recorded as the degree of alveolar bone loss as a percentage of root length.

If indicated based on the presence of periapical lucencies or periodontitis, affected mandibular first molar teeth were extracted in their entirety, or sectioned with a high-speed handpiece in a manner to preserve the crown of the tooth. Extracted teeth were stored in 10% neutral buffered formalin until analyzed with one or more of the following methods. (1) High-resolution micro-CT of undecalcified teeth. (2) Light microscopy using routine hematoxylin and eosin (H&E) stained histological slides of decalcified teeth. (3) Light microscopy of immunohistochemically (IHC) stained slides of decalcified teeth. (4) Light microscopy of undecalcified ground sections.

### Micro-CT Evaluation

Extracted teeth were placed in a 50 mm Falcon tube along with sufficient storage medium to cover the teeth, and secured using cotton wool prior to being scanned in a high-resolution 3D X-ray microscope (ZEISS Xradia Versa 520, Carl Zeiss Microscopy, Thornwood, NY) using a slice thickness of 24 μm. Image data was imported in DICOM format into dedicated radiology imaging software (Carestream Vue PACS, Carestream Health, Rochester, NY) for viewing on a dedicated image viewing workstation by a board-certified veterinary radiologist (IP), and a dentistry and oral surgery resident in training (KKN) using a center of 27,000 arbitrary brightness units with a range of 45,000 arbitrary brightness units. Multiplanar reconstructions (MPR) in the transverse, sagittal and dorsal planes were utilized as needed. Conclusions were established by consensus. Additional enamel fissures that were detected on micro-CT were confirmed grossly by application of plaque-disclosing solution (Reveal, Henry Schein, Melville, NY).

### Light Microscopy of Undecalcified Ground Sections

Teeth were submitted for evaluation with ground sections if they had evidence of enamel fissures on micro-CT. Multiplanar reconstructions of the micro-CT volumes of the extracted teeth were used to plan planes of interest through enamel fissures. Teeth were sectioned with a thin, 22 mm-diameter diamond disc (Diamond Disc 22 mm Double Sided—HP, iM3Vet, Vancouver, WA) 1 mm parallel to the plane of interest, while ensuring enough tissue of interest remained in both “halves” for analysis. The portion of the tooth with the plane of interest was submitted to the Hard Tissue Research Laboratory at University of Minnesota's School of Dentistry for ground sectioning, along with gross and CT images illustrating the plane of interest. The remainder of the tooth was reserved for decalcification, H&E staining and IHC. Specimens were dehydrated with a graded series of alcohols for 9 days. Following dehydration, the specimens were infiltrated with a light-curing embedding resin (Technovit 7200 VLC, Kulzer, Wehrheim, Germany) for 20 days with constant shaking at normal atmospheric pressure, before the resin was polymerized by 450 nm light with the temperature of the specimens never exceeding 40°C. The specimens were then prepared along planes of interest by the cutting/grinding method as previously described (15, 16). Briefly, the specimens were cut to a thickness of 150 μm on an EXAKT cutting/grinding system (EXAKT Technologies, Oklahoma City, OK). Following this, specimens were polished to a thickness of 35–50 μm using a series of polishing sandpaper discs from 800 to 4,000 grit using an EXAKT micro-grinding system (EXAKT Technologies, Oklahoma City, OK) followed by a final polish with 0.3 μm alumina polishing paste. The slides were stained with Stevenel's blue and counterstained with Van Gieson's picro fuchsin and evaluated by the same pathologists (AC, SR). Stevenel's blue stains cells and extracellular structures in gradations of blue, while leaving mineralized tissues unstained. Van Gieson's picro-fuchsin stains collagen fibers green or green-blue, bone orange or purple, osteoid yellow-green, and muscle fibers blue to blue-green (17).

### Light Microscopy and Immunohistochemistry of Decalcified Specimens

Remaining “halves” from teeth submitted for ground sections and all other extracted affected teeth were decalcified in 22% formic acid until suitable for sectioning, processed using routine histologic processing techniques, embedded in paraffin, and cut at 4-μm thickness. Sections were stained with H&E. Immunohistochemistry for amelogenin was performed on an automated IHC staining system at room temperature (BOND-MAX, Leica Biosystems, Buffalo Grove, IL) to determine if the abnormal tissue contained detectable amelogenin and thus confirm if there is a component of enamel. Tissue for positive controls were obtained from developing tooth germ from a stillborn full-term canine fetus. Formalin-fixed, paraffin-embedded sections were deparaffinized, rehydrated, and subsequently blocked with 3% hydrogen peroxide. Antigen retrieval was performed using the Bond Epitope Retrieval Solution 2 (Leica Biosystems, Buffalo Grove, IL) for 10 min. Sections were incubated with anti-mouse amelogenin [Amelogenin Antibody (F-11), Santa Cruz Biotechnology, Dallas, TX] for 15 min. Slides were then incubated with secondary antibody (PowerVision Poly-HRP Anti-Mouse IgG, Leica Biosystems, Buffalo Grove, IL) for 10 min. The slides were then incubated with a polymer (Bond Polymer Refine Detection Kit, Leica Biosystems, Buffalo Grove, IL) for 30 min for visualization. Negative control slides consisted of the tissue of interest undergoing the same process as the positive control and test slides without the primary antibody. Positive and negative controls were adequate.

Representative histologic sections were selected and mounted. Micro-CT MPR images approximating the sections were reconstructed for comparison. Histologic sections were evaluated by a board-certified veterinary pathologist (AC) and anatomic pathology resident in training (SR) and conclusions were established by consensus.

## Results

Six dogs with malformations of the mandibular first molar teeth were identified, representing a total of 11 affected mandibular first molar teeth. In 5 dogs, the mandibular first molar teeth were bilaterally affected, while in 1 dog, the left mandibular first molar tooth was absent at the time of diagnosis. Signalment data for all patients are displayed in Table 1. The dogs were aged from 0.4 to 14 years (median = 5 years) at the time of diagnosis and weighed 1.7–6 kg (median = 4.09 kg). The medical history was unremarkable with no pre-existing comorbidities in all patients. None of the clients could recall episodes of illness in the patient prior to eruption of the permanent dentition or in the dam during gestation or parturition. Point-of-care bloodwork or serum biochemical profiles with complete blood count was considered unremarkable in all patients.

**Table 1:** Summary data for 6 dogs with developmental abnormalities of the mandibular first molar teeth.

**Dog**	**Age at diagnosis**	**Sex**	**Weight (kg)**	**Breed**	**Other developmental dental abnormalities**
1	5 m	Male castrated	6	Cavalier King Charles Spaniel	Similar lesions on pulp floor of right and left maxillary fourth premolar teeth Pulp stones in all 4 first premolar teeth and left mandibular canine tooth
2	5 y	Female spayed	4 .7	Poodle Mix	None
3	5 y	Female spayed	1 .7	Shih Tzu Mix	None
4	9 y	Male castrated	4 .4	Pomeranian	None
5	14 y	Female spayed	3 .8	Miniature Pinscher	None
6	5 y	Male castrated	3 .7	Mixed	Similar lesions on pulp floor of right and left maxillary fourth premolar teeth

**Figure 1:**
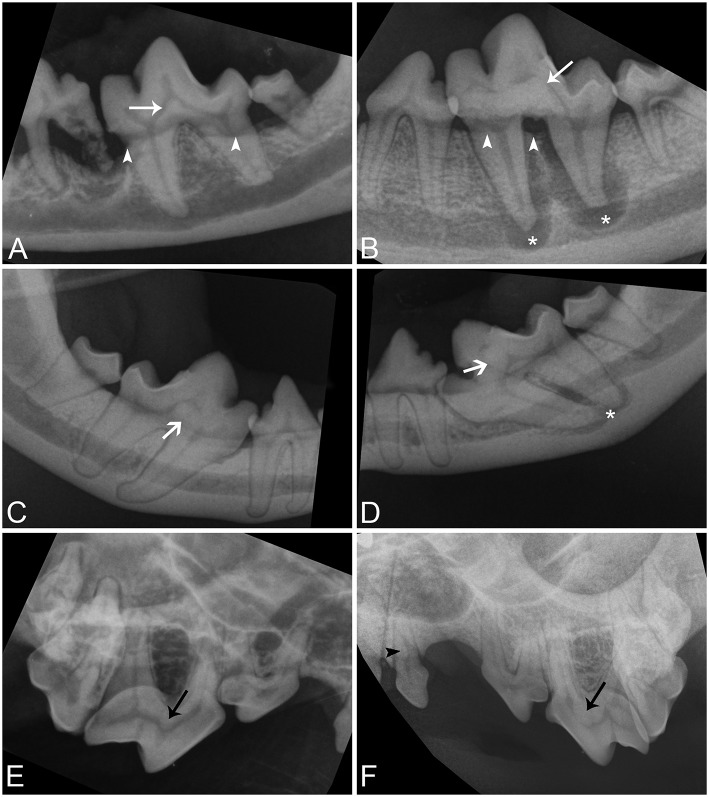
Intraoral radiographic images of affected teeth. **(A,B)** Parallel radiographs of the left mandibular first molar tooth from dog 5 and 1, respectively, demonstrating the variation in size of abnormal tissue on the floor of the pulp chamber (white arrows). **(C,D)** Parallel radiographs of the right and left mandibular first molar teeth from dog 3, respectively, demonstrating asymmetry in the location of lesions and conformational abnormalities (white open arrows). Both mandibular first molar teeth are mesioverted and under-erupted bilaterally. The abnormal tissue of tooth is centered on the floor of the pulp chamber in the right mandibular first molar tooth but is centered on the mesial wall of the pulp chamber in the left mandibular first molar tooth. **(E,F)** Bisecting angle radiographs of the caudal right maxilla of dog 6 and the caudal left maxilla of dog 1 demonstrating abnormal tissue on the floor of the pulp chambers of the maxillary fourth premolar teeth (black arrows), and a pulp stone in the left maxillary first premolar tooth in dog 1 (black arrowhead). Periapical lucencies (**B,D**—white asterisk), and alveolar bone loss (**A,B**—white arrowheads) affect some teeth.

**Figure 2:**
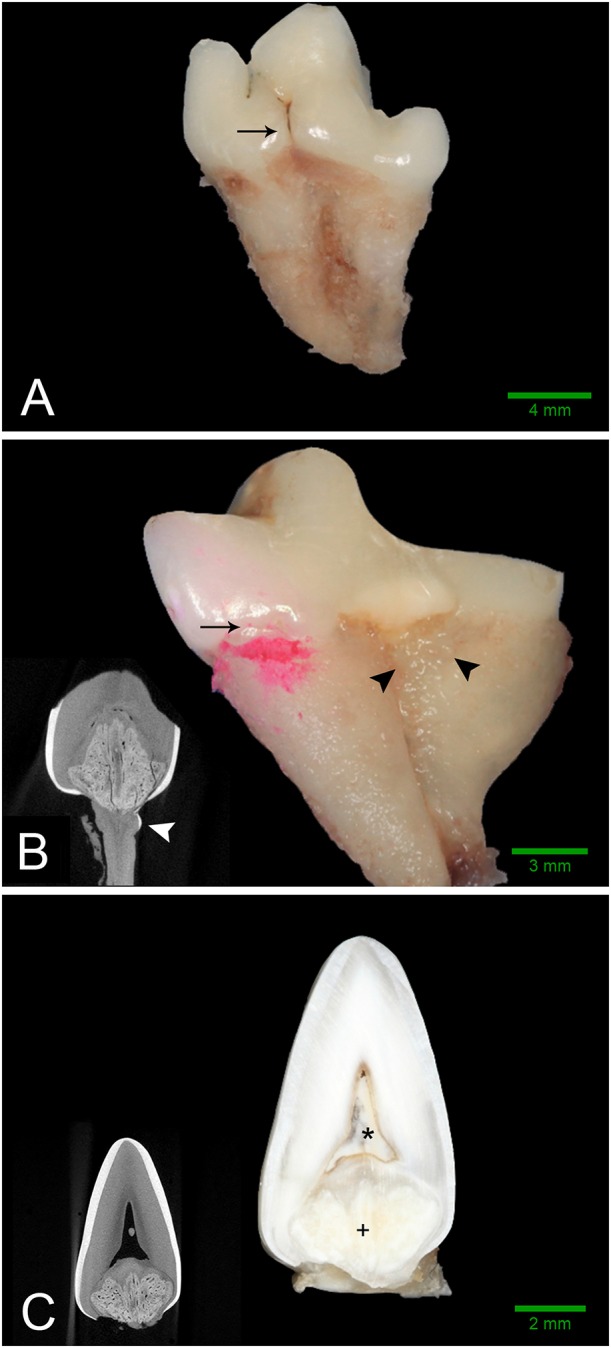
Gross features of affected teeth. Scale bars are for gross images only. **(A)** Buccal aspect of the left mandibular first molar tooth from dog 3 demonstrating an obvious enamel fissure on the buccal aspect of the tooth (black arrow), as well as severely convergent roots. **(B)** Mesio-lingual aspect of the right mandibular first molar tooth from dog 2. Disclosing solution has been used to highlight a subtle enamel fissure on the mesiolingual aspect of the tooth (black arrow). Irregular, thin, ectopic enamel extends down the radicular surface in the region of the furcation (black arrowheads). Severely convergent roots and abrasion with tertiary dentin are also evident. (**B**, inset) Transverse micro-CT reconstruction at the level of the furcation confirming the presence of hyperattenuating material on the root surface consistent with ectopic enamel (white arrowhead). **(C)** Bucco-lingual section through the left mandibular first molar tooth of Dog 1. A solid mass of abnormal hard tissue (+) is located on the floor of the pulp chamber. The pulp chamber is denoted with an (*). (**C**, inset) Transverse micro-CT reconstruction along the same plane as the section.

Intraoral radiographs were available for all 11 mandibular first molar teeth. The primary finding in affected teeth was the presence of abnormal tissue of dentin opacity of varying size within the crown (Figure 1). In 10 of 11 teeth, the abnormal tissue was centered in the floor of the pulp chamber, while in the remaining tooth, the abnormal tissue instead appeared to be centered on the mesial wall of the pulp chamber. Clinical and radiographic features of affected mandibular first molar teeth are listed in Table 2. Root convergence or parallelism was noted in 6 of 11 teeth and root dilaceration was noted in 3 of 11 teeth. Initial gross examination of the tooth crowns identified enamel fissures close to the cementoenamel junction on the buccal aspect of the crown in 2 of 11 teeth (Figure 2A).

**Table 2:** Clinical and radiographic characteristics of mandibular first molar teeth with developmental abnormalities.

**Dog**	**Tooth**	**Root conformation**	**Periapical lucency**	**Periodontitis (% of alveolar bone loss)**	**Number of enamel fissures**	**Enamel fissure(s) location**	**Contact betweenbreak abnormal tissue and:**	**Channel communication with:**	**Ectopic Enamel**
1	Left	Parallel	Yes	<25%, furcation exposure	5+^*^	Mesial, buccal, lingual	Enamel fissure, furcation	Pulp, enamel fissure, furcation	Yes
	Right^†^	Parallel	No	No					
2	Left^#^								
	Right	Convergent	Yes^‡^	<25%	5+^*^	Mesial, buccal, lingual	Enamel fissure, furcation	Pulp, enamel fissure, furcation	Yes
3	Left	Convergent	Yes	No	1	Buccal	Pulp, enamel fissure	Pulp	No
	Right	Parallel, dilaceration	No	<25%	0	N/A	None	Pulp	No
4	Left	Divergent, dilaceration	Yes^§^	>50%, furcation exposure	0	N/A	Furcation	None	No
	Right	Divergent, dilaceration	Yes^§^	>50%, furcation exposure	0	N/A	Furcation	None	No
5	Left^†^	Divergent	No	25–50%					
	Right	Divergent	No	>50%, furcation exposure	0	N/A	None	None	No
6	Left	Convergent	Yes	<25%	1	Buccal	Enamel fissure, furcation	None	No
	Right^†^	Divergent	No	No					

* *Detected with micro-CT and confirmed grossly*.

# *Absent*.

† *Not extracted*.

‡ *Concurrent pulp exposure due to abrasion*.

§ *Concurrent severe generalized periodontitis*.

Full-mouth radiographs revealed additional teeth with pulpal opacities in 2 of 6 dogs. In both of these dogs abnormal tissue of dentin opacity were present in the floor of the pulp chamber of the maxillary fourth premolar teeth, similar in appearance and location to the lesions located in the mandibular first molar teeth. Additionally, one of these dogs also had focal tissue of dentin opacity located within the pulp cavity of an additional five teeth. In all four first premolar teeth, these opacities were located within the root canal, while in the left mandibular canine tooth, it was located at the cervical level.

Eight out of the 11 mandibular first molar teeth were extracted due to the concurrent presence of periapical lucencies or periodontitis. In 3 dogs, both affected mandibular first molar teeth were extracted at the time of diagnosis, whereas in 2 dogs, only 1 affected tooth was extracted. Dog 1 did not show any clinical or radiographic abnormalities associated with either tooth at the time of diagnosis at 5 months of age but developed periapical lucencies and periodontitis of the left mandibular first molar tooth at 20 months of age. This tooth was extracted, and the client opted for ongoing radiographic monitoring of the right mandibular first molar tooth. Historical intraoral radiographs were available for dog 4; the abnormalities were diagnosed at 9 years of age, but previous intraoral radiographs were available from 3 years of age. On these radiographs, the lesions were present and of similar size to the radiographs at 9 years of age. There was mild alveolar bone loss affecting both mandibular first molar teeth at 3 years of age but no radiographic evidence of apical periodontitis. Extracted teeth were submitted for further evaluation as detailed in Table 3.

**Table 3:** Summary of investigations performed on 8 extracted mandibular first molar teeth with developmental abnormalities.

**Dog**	**Tooth**	**Outcome**	**Micro-CT**	**Ground sections**	**H&E**	**Amelogenin**
1	Left	Extracted	✓	✓	✓	✓
	Right	Not extracted	–	–	–	–
2	Left	Absent	–	–	–	–
	Right	Extracted	✓	✓	✓	✓
3	Left	Extracted	✓	✓	✓	✓
	Right	Extracted	✓	–	✓	✓
4	Left	Extracted	✓	✓	✓	✓
	Right	Extracted	✓	–	✓	✓
5	Left	Not extracted	–	–	–	–
	Right	Extracted	✓	–	✓	✓
6	Left	Extracted	✓	✓	✓	✓
	Right	Not extracted	–	–	–	–

**Figure 3:**
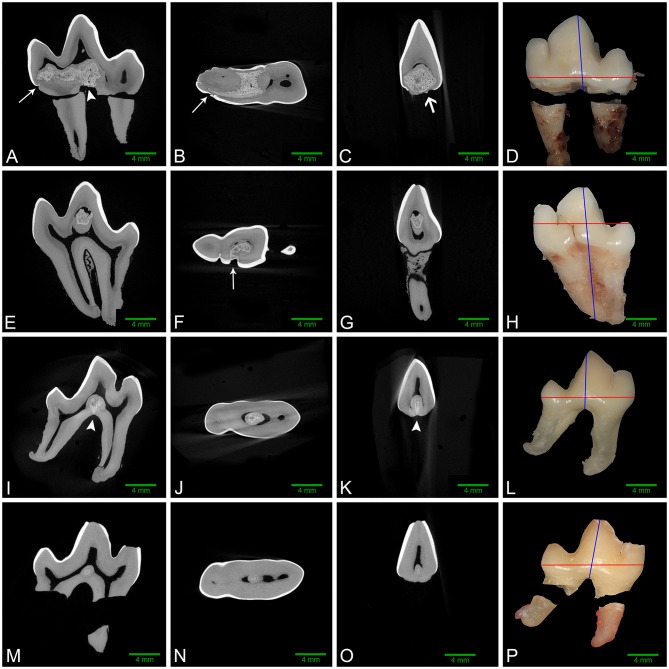
Left to right: sagittal, dorsal, and transverse micro-CT reconstructions along with gross images of the buccal aspect of affected mandibular first molar teeth. Red and blue lines on the gross images denote the plane of dorsal and transverse sections, respectively. **(A–D)** Left mandibular first molar tooth from dog 1. The abnormal tissue occupies a large region of the pulp chamber floor and communicates with multiple enamel fissures (white arrows), as well as the furcation (white arrowhead). A small focus of ectopic enamel is also visible (open white arrow), and a free-floating pulp stone is present in the central pulp horn. **(E–H)** Left mandibular first molar tooth from dog 3. Abnormal tissue occupies a large area of the central pulp horn and communicates with a large enamel fissure on the buccal aspect of the crown (white arrow), with no contact with the furcation. **(I–L)** Right mandibular first molar tooth from dog 4. The lesion in the central pulp chamber floor contacts the furcation (white arrowheads), but not the coronal enamel. **(M–P)** Right mandibular first molar tooth from dog 5. The small lesion on the floor of the pulp cavity has no identifiable contact with enamel or the radicular surfaces.

**Figure 4:**
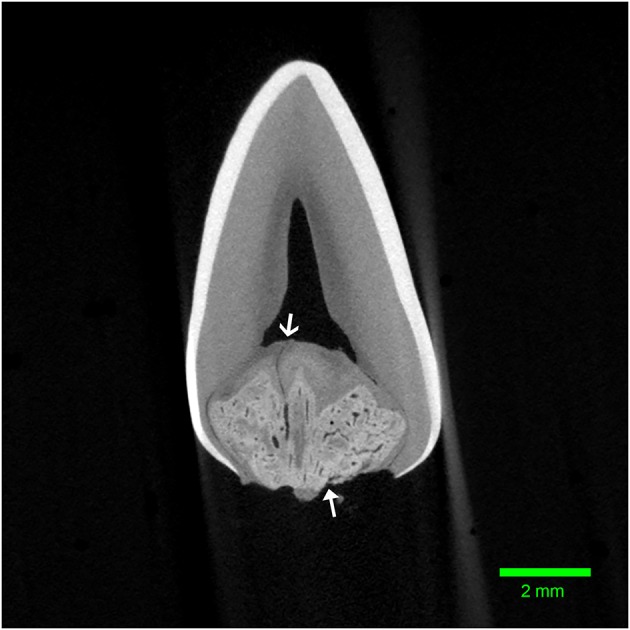
Transverse micro-CT reconstruction through the furcation of the left mandibular first molar tooth from dog 1. Note the corono-apical oriented channel connecting the abnormal tissue to the pulp (open arrow). The entrance to another channel can be seen communicating with the furcational periodontium (closed arrow).

Micro-computed tomography was performed on all extracted teeth. The abnormal tissue was intermediate in attenuation between that of enamel and dentin, and appeared heterogenous (Figure 3). It contacted the enamel in 4 of 8 teeth on micro-CT and in the areas of contact, the enamel was frequently discontinuous or indented, resulting in an enamel “fissure.” As described previously, this was obvious grossly in 2 of these teeth, both of which had only 1 fissure. In the remaining 2 teeth, multiple small fissures were identified on micro-CT that required magnification and disclosing solution to visualize grossly (Figure 2B). These fissures were located on the mesial, buccal and lingual aspects close to the cementoenamel junction. The abnormal tissue extended to the furcation in 5 of 8 teeth while contact with both the enamel and the furcation was identified in 3 of 8 teeth. In 2 teeth, the abnormal tissue did not appear to contact either the external enamel or the furcation. Channels measuring ~20–40 μm in diameter traversed the abnormal tissue in all teeth (Figure 4). The channels were predominantly oriented in a corono-apical direction. Communication of the channels with the pulp, oral cavity and/or furcation could be identified in 4 of 8 teeth. Hyperattenuating tissue consistent with ectopic radicular enamel extended into the furcation in 2 of 8 teeth (Figures 2B, 3C).

**Figure 5:**
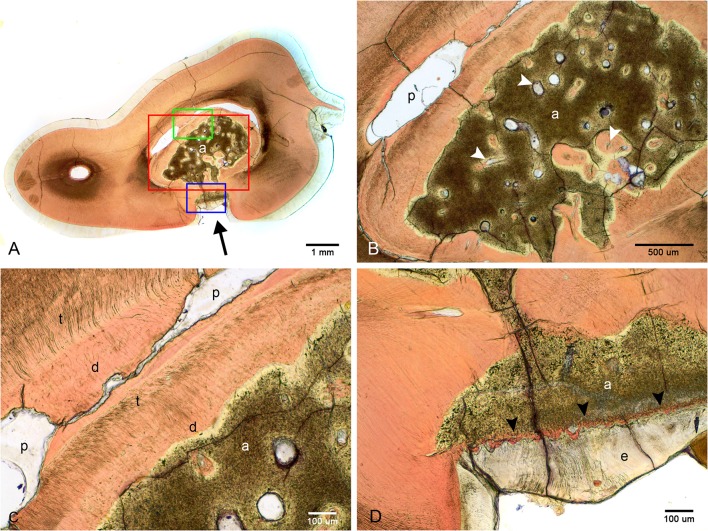
Ground sections of the left mandibular first molar tooth from dog 6. **(A)** Low magnification of a mid-coronal dorsal section through the abnormal tissue and enamel fissure (black arrow). **(B)** Medium magnification of the area marked by the red rectangle in **(A)**. Several channels traversing the abnormal tissues (white arrowheads), some surrounded by dentin-like material. **(C)** High magnification of the area marked by the green rectangle in **(A)**. Disorganized dentin is visible lining the abnormal tissue and the pulp. **(D)** High magnification of the area marked by the blue rectangle in **(A)**, demonstrating a thin band of dentin-like tissue separating the abnormal tissue and enamel (black arrowheads). a, abnormal tissue; d, disorganized dentin; t, tubular dentin; e, enamel; p, pulp.

Examination of undecalcified ground sections was performed on 4 of 8 extracted teeth with enamel fissures. An additional tooth with contact between the abnormal tissue and furcation also had ground sections performed. Gross examination of the interior of the lesions was performed on all 5 teeth submitted for ground sections, which revealed a solid mass of abnormal hard tissue (Figure 2C). In 3 of 5 teeth, small amounts of alveolar bone were inadvertently included in the sample (not shown). The lesion was trimmed out in one sample leaving the lesions of 4 teeth available for evaluation. On ground sections, normal-appearing dentin, cementum, and bone was stained orange by Van Gieson's picro-fuschsin. The abnormal tissue was irregularly lined but well-demarcated, with yellow-gray staining (Figure 5). In comparison, external enamel had minimal stain uptake. The abnormal tissue was disorganized and lacked identifiable enamel rods and Hunter-Schreger bands, both of which were noted to be present in the external enamel of evaluated teeth. Other histological features such as enamel tufts, enamel spindles, enamel lamellae, and striae of Retzius were not identified in either the external enamel or the abnormal tissue in any of the sections examined. In areas of contact with the external enamel, the abnormal tissue either appeared discontinuous from the external enamel with an abrupt transition, or were separated by a thin band of dentin-like tissue (Figure 5D). While the abnormal tissue itself did not have identifiable dentinal tubules, it appeared to be surrounded by a layer of predominantly disorganized dentin, some areas of which demonstrated features consistent with globular dentin (not shown). The abnormal tissue appeared to be distinct from this surrounding dentin. Additionally, there were numerous channels within the abnormal tissues, some of which were surrounded by small islands of material of similar color and texture to dentin (Figure 5B).

**Figure 6:**
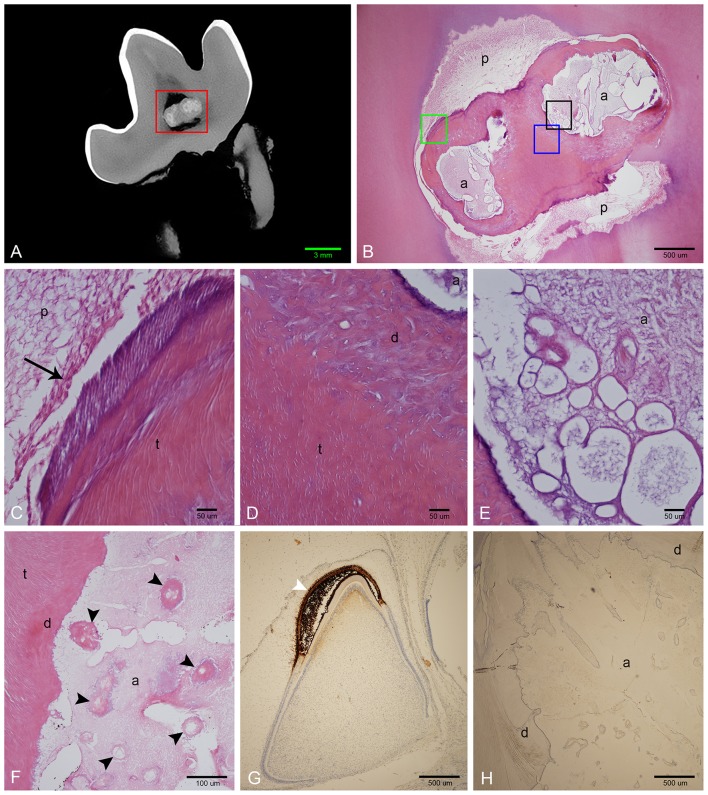
Histological features of affected teeth on decalcified sections. **(A)** Sagittal micro-CT of the right mandibular first molar tooth from dog 3. **(B)** Low magnification H&E stain of a section approximating the area marked by the red rectangle in **(A)**. The abnormal tissue is composed of small amounts of acellular eosinophilic matrix, yet corresponding areas on the micro-CT image are relatively highly attenuating, indicating that the bulk of material has been lost during decalcification. **(C)** High magnification of area denoted by green rectangle in **(B)**. Vital pulp lined with odontoblasts (black arrow). **(D)** High magnification of area denoted by blue rectangle in **(B)**. Disorganized dentin lines the abnormal tissue, transitioning to tubular dentin further away. **(E)** High magnification of area denoted by black rectangle in **(B)**, showing detail of acellular matrix with basophilic staining at the periphery. **(F)** High magnification of an area of abnormal tissue from the left mandibular first molar tooth from dog 1. Multiple islands of hypereosinophilic material resembling dentin with central channels can be seen (black arrowheads) within the mostly eosinophilic acellular matrix. **(G)** Low magnification of positive control slide (tooth germ of a full-term canine fetus) treated with anti-amelogenin antibody showing strong positive reaction in the enamel layer of a developing tooth germ in bell stage (white arrowhead). **(H)** Low magnification of a transverse section of the left mandibular first molar tooth in dog 1 stained with anti-amelogenin antibody, showing no positive reaction. a, abnormal tissue; d, disorganized dentin; t, tubular dentin; p, pulp.

Demineralized sections were examined on all extracted teeth (Figure 6). The abnormal tissue was characterized by flocculent to fibrillar, acellular matrix with numerous, variably sized, bulbous, clear spaces. This matrix was lightly eosinophilic, with slight basophilic staining at its borders (Figures 6E,F). Intermittently but fairly regularly positioned in this matrix, were hypereosinophilic, acellular, irregular islands of material with a seeming central vascular channel and variable thin basophilic lines that resembled reversal lines (Figure 6F). This material had a similar appearance to the disorganized dentin that was lining this locally extensive affected area (Figure 6D). Areas with clear spaces correlated with areas of higher attenuation on micro-CT indicating a higher mineral component than dentin, which was removed during the decalcification process (Figures 6A,B). Immunohistochemistry of this amorphous matrix for amelogenin was negative in all samples (Figure 6H).

## Discussion

Odontogenesis is a complex physiological process involving the expression of multiple genes and bidirectional signaling between ectoderm and ectomesenchyme (18). Heritable, systemic, or local insults that disrupt this process may result in morphological abnormalities of the teeth (2). Developmental abnormalities of the dentition previously reported in dogs include enamel hypoplasia, dentinogenesis imperfecta, developmental tooth discoloration, gemination, fusion, concrescence, dens invaginatus, enamel pearls, supernumerary roots, hyponumerary roots, root dilaceration, regional odontodysplasia, and dentin dysplasia (1, 2, 19, 20). In this study, we provide an in-depth description of a previously reported but incompletely characterized developmental abnormality that affects mandibular first molar teeth in dogs: the presence of abnormal mineralized tissue surrounded by disorganized dentin within the tooth.

While the acellular matrix of the abnormal tissue observed on decalcified sections was suggestive of enamel matrix, other histologic features of enamel were not identified. Ground section features of normal enamel in humans include enamel prisms, Hunter-Schreger (H-S) bands, striae of Retzius, enamel tufts, enamel spindles, and enamel lamellae (21, 22). While enamel prisms and H-S bands were identifiable in the external enamel in the teeth reported here, none of the histologic features of enamel were identifiable in the abnormal tissue. In contrast, visible enamel prisms (23, 24) and H-S bands (23, 25, 26) have been reported in the ectopic internal enamel in cases of dens invaginatus in people. Once the abnormal tissue was demineralized, the remaining eosinophilic matrix was devoid of identifiable enamel rods. In contrast, identifiable enamel matrix has previously been described in invaginated enamel in human dens invaginatus (27, 28), as well as in cases of amelogenesis imperfecta in dogs (29). Immunohistochemistry with anti-amelogenin antibody did not detect amelogenin in the residual matrix. A positive result would have provided evidence that the abnormal tissue was dysplastic, aprismatic enamel, but a negative result does not rule this out, as amelogenin, if initially present, could have been subsequently degraded during enamel maturation. Likewise, if the abnormal tissue was indeed enamel, but was severely dysplastic, then enamel prisms, H-S bands, and identifiable enamel matrix may not be present.

Several features of the abnormal tissue in the cases reported here suggest that it originates from the dental papilla rather than the enamel organ. Firstly, smaller lesions were surrounded by dentin and were isolated from the external enamel and the radicular surface. Secondly, in areas where the abnormal tissue and the external enamel did come into contact, they appeared distinct, suggesting that these two tissues arise separately. Conversely, the abnormalities described here had histologic features that differed from tissues expected to arise from the dental papilla or dental sac. For example, the abnormal tissue described here was acellular, lacked identifiable dentin tubules, and had different staining characteristics to dentin, mature bone, or cementum when stained with Van Gieson's picro-fuschsin. It was also clearly demarcated from surrounding dentin, which tended to be disorganized and incompletely mineralized, suggesting that the abnormal tissue stimulates dentin formation via irritation to the pulp rather than via induction.

Based on these results, it was not possible to definitively identify the nature of the abnormal tissue observed as either dentin or enamel. If the abnormal tissue is indeed enamel, it does not appear to arise from invaginations of the enamel organ. The authors are not aware of any dental developmental abnormality where internal deposits of enamel arise without invagination of the enamel organ during odontogenesis. Therefore, potential candidates such as enamel, dentin, cementum and bone should still be considered and further histological and immunohistochemical investigations are required to determine the developmental origin of the abnormal tissue.

Despite several previous reports classifying similar developmental abnormalities of the mandibular first molar teeth as dens invaginatus in dogs, we identified clinical, ultrastructural and histological features inconsistent with this condition as described in humans. Dens invaginatus is a relatively common developmental abnormality that has been methodically described in humans (12, 30–33). It is characterized by enamel-lined cavities in the tooth structure caused by invaginations of the enamel organ or Hertwig's epithelial root sheath during odontogenesis (3, 30, 34), whereas in the cases described here, the lesions consisted of a “mass” of abnormal tissue, rather than a cavity. Moreover, the abnormal tissue does not appear to arise from invagination of the enamel organ, and we were unable to confirm that the abnormal tissue was enamel. While the enamel “fissures” reported here grossly resemble enamel fissures or pits denoting the point of invagination in human dens invaginatus, there are several differences. Firstly, the presence of enamel fissures was inconsistent, and they were only present when the abnormal tissue contacted the external enamel, whereas in human dens invaginatus, the fissure or pit denotes the point of origin of the invagination process, and small lesions would be located at a lingual, incisal, or occlusal pit (30, 31), rather than surrounded by dentin as in these cases. Secondly, fissures were located close to the cementoenamel junction, whereas they typically occur on the occlusal surface in human dens invaginatus of molar teeth (31). Thirdly, in 2 teeth, more than 5 fissures were identified, all contacting the same mass of abnormal tissue, whereas multiple invaginations in human dens invaginatus are rare, with up to 3 invaginations having been reported, with each fissure typically leading to a separate invagination (30, 35, 36). Furthermore, based on existing veterinary literature, abnormalities that have been previously attributed to “dens invaginatus” appear to have a predilection for bilateral involvement of the mandibular first molar teeth (4), whereas dens invaginatus as reported in humans is most commonly reported to affect the maxillary second incisor tooth (37, 38), bilateral involvement is only reported in 24–43% of cases (37–40), and involvement of mandibular teeth is rare (12, 37, 38, 40, 41).

Likewise, the morphology of the mandibular first molar teeth in this study differed from that reported for human enamel pearls. Aggregates of ectopic radicular enamel are classified into either enamel pearls, defined as discrete protuberances of enamel on the radicular surface (42), or cervical enamel extensions, finger-like projections of enamel that extend from the cementoenamel junction toward the furcation in molar teeth (3). The ectopic enamel identified on micro-CT in two of teeth in this study was continuous with the coronal enamel, and was flattened in appearance, which resembles cervical enamel extensions as described in humans. Furthermore, enamel pearls in humans are more prevalent in maxillary molar teeth, and bilateral involvement appears to be rare (13), whereas cervical enamel extensions are reportedly more common in mandibular molars (43). Enamel pearls have been previously reported in a maxillary fourth premolar of a German shepherd dog, a mandibular first molar in a Maltese dog, and in both mandibular first molar teeth in an Irish setter (1, 5). Interestingly, the teeth affected in these cases are the same as the teeth involved in this study, and all the affected mandibular first molar teeth in these previous cases demonstrated root convergence. As imaging studies were not performed in these previous reports, the possibility that these teeth may have been examples of the malformations described in this study cannot be ruled out. However, as far as the authors are aware, neither enamel pearls nor cervical enamel extensions in humans are associated with internal abnormal tissue deposits similar to those described in this study. It is also unclear if cervical enamel extensions and enamel pearls occur without the presence of abnormal hard tissue deposits in dogs. Thus, the use of these terms may be inappropriate for these abnormalities in dogs.

**Figure 7:**
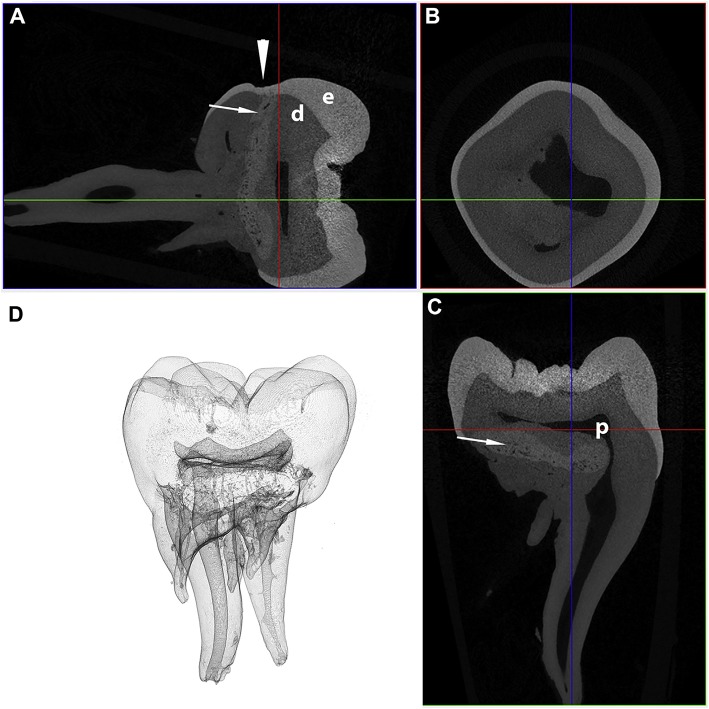
Transverse **(A)**, coronal **(B)**, and sagittal **(C)** micro-CT images in bone algorithm and a 3D volume rendered reconstruction **(D)** of a human mandibular first molar with MIM. Note the abnormal tissue with attenuation characteristics between dentin and enamel at the cervical region (white arrows), as well as a depression in the enamel, known as a cervical constriction, at the level where the abnormal tissue contacts the dentinoenamel junction (white arrowhead) [from: Brusevold et al. (47)—reproduced with permission].

On the contrary, the micro-CT appearance of the abnormal tissue resembles descriptions of abnormal dentin in dentinogenesis imperfecta Type II (44), or the abnormal tissue in molar-incisor malformation (MIM) (45–48). However, dentinogenesis imperfecta, an autosomal dominant abnormality resulting in abnormal dentin formation, would be expected to involve all teeth (3), while the distribution of the lesions in the cases described here is best described as symmetric and multi-quadrant. Molar-incisor malformation is a recently described developmental dental abnormality in humans characterized by the presence of abnormal hard tissue in the permanent maxillary and mandibular first molar teeth, and occasionally the maxillary first incisor teeth (45–49). In contrast with dentinogenesis imperfecta, MIM shares multiple clinical features with the cases described here, including the symmetrical distribution (50), the location of the abnormal tissue at the cervical region with deviation of the surrounding pulp chamber (45–48), depressions or fissures in the enamel at the cervical level (47, 50, 51), and presence of root malformations [(45–49, 51–54); Figure 7]. The abnormal tissue in MIM is believed to originate from the dental papilla (46) or dental follicle (45, 46), with most authors describing the tissue as either osteodentin-like (52, 54), globular dentin (47, 50), or dysplastic dentin (53). This hypothesis is further supported by strong expression of dentin sialoprotein and osteocalcin on IHC in MIM (46). One author also reported the presence of odontoblast-like cells in the abnormal tissue (46). In contrast, while the location of the abnormal tissue in our study suggests that the abnormal tissue originates from the dental papilla, we did not find any histological features consistent with dentin. Based on these findings, it appears that these developmental abnormalities in mandibular first molar teeth in dogs bears close resemblance to MIM in humans, yet with some histological differences.

No matter the terminology used to describe these lesions in dogs, the large proportion of affected teeth with periapical lucencies or periodontitis emphasizes their clinical significance. Several features of this condition may have contributed to this. The enamel fissures act as plaque-retentive surfaces that may increase the risk of caries lesion formation, particularly as the enamel fissures are associated with contact of the less-mineralized, and thus more caries-prone, abnormal tissue with the coronal surface. The ectopic furcational enamel has the potential to contribute to the development of periodontitis due to the inability of periodontal ligament to adhere to enamel (3). The channels traversing the abnormal tissue, in some cases communicating with the furcation and enamel fissures, are large enough to allow bacterial translocation into the pulp from areas affected by periodontitis, caries, or directly through fissures in the enamel. The nature of these channels is uncertain and do not appear to be lined with endothelium, nor filled with blood. Similar channels have previously been reported in MIM (45, 47–49). Although successful endodontic treatment of MIM (49) and mandibular first molar teeth in dogs with furcational abnormalities similar to those described in this study (6) have been reported, it may be difficult to eliminate bacteria in these additional channels during endodontic treatment, possibly leading to an increased risk of treatment failure in these teeth. Furthermore, the abnormal tissue may act as a barrier for instrumentation, and the presence of occult ectopic radicular enamel may predispose affected teeth to periodontitis. Therefore, extraction of affected teeth may be the most reliable treatment.

In 2 of 6 dogs, full-mouth radiographs identified other radiopacities within the crowns of both maxillary fourth premolar teeth, also known as the maxillary carnassial teeth. Radiographically, the changes in the maxillary carnassial teeth resemble those in the mandibular carnassial teeth, and the authors opine that these teeth are likely affected by the same disease process. While radiopacities within the first premolar teeth and one canine tooth of 1 of these 2 dogs were also noted, the location of the opacities differed from that of the maxillary fourth premolars and the mandibular first molars. These abnormalities were located centrally within the pulp cavity, and thus resemble free pulp stones as described in the human literature (55). While mandibular first premolar teeth have previously been reported to be affected in two dogs with abnormalities of the mandibular first molar teeth similar to those reported here (11), these other teeth in our cases were not extracted due to the lack of clinical reasons to do so, and thus were not subjected to the same rigorous evaluation as the mandibular first molar teeth. Therefore, it cannot be confirmed if the teeth other than the mandibular first molar teeth are affected by the same disease process, and it remains possible that other teeth may be affected other than the mandibular first molar teeth. It also remains unclear whether prior reports of dens invaginatus in dogs involving maxillary molar (56, 57), or canine teeth (58) differ from the abnormalities reported here. Evaluations of teeth other than mandibular first molars with abnormal radiopaque structures in the crown should involve micro-CT and histology of decalcified and non-decalcified specimens to determine if other teeth can be affected with this condition.

The consistent involvement of specific teeth in these dogs is suggestive of a noxious stimulus at a specific time point during odontogenesis. Although adverse medical events at any time point during gestation or in the immediate post-natal period were not reported in any of the dogs in this study, it is worth noting that information from the prenatal and antenatal periods may be unreliable if there was a change in ownership of the dog between parturition and presentation. Additionally, all the dogs in this study were small breed dogs, and dams of small or miniature breeds of dogs may be predisposed to developing dystocia (59, 60). Similar malformations have previously been reported in young dogs with uremia (11), and MIM is recognized as being associated with adverse medical events antenatally or in the immediate post-natal period (45–49, 51–54). Thus, adverse medical events occurring during gestation, parturition or antenatally cannot be completely ruled out in the dogs in this study. Small breed dogs reportedly have mandibular first molar teeth that are disproportionately large relative to the jaws (61, 62), and this tooth is reported to have a higher prevalence of root dilaceration in this patient class (4). Therefore, it is also possible that this anatomical quirk may contribute to a noxious stimulus preferentially affecting this tooth in some way. Further investigations are recommended to investigate the effect of breeding for small patient size on the dentition in dogs.

We conclude that the dental abnormalities described here correspond to a previously unrecognized developmental abnormality in dogs that affect the mandibular, and possibly the maxillary, carnassial teeth. Further investigations are required to determine the mode of formation, origin of the abnormal tissue, factors associated with development, and if teeth other than mandibular first molars can be affected. In the absence of a known etiology, and considering the morphological and histological differences from human dens invaginatus, enamel pearls and MIM, the authors recommend that malformations in both mandibular and maxillary carnassial teeth with the features described here be simply referred to as carnassial-tooth malformations until further information is available.

## Data Availability Statement

All datasets generated for this study are included in the article.

## Ethics Statement

The use of archived diagnostic material or review and data collection from medical records of client-owned animals for the purposes of this study was approved by Cornell University's Veterinary Clinical Studies Committee and was considered exempt from review by Cornell University's Institutional Animal Care and Use Committee. Written informed consent was obtained from the owners for the participation of their animals in this study.

## Author Contributions

KN drafted the manuscript, designed the study, collected samples, analyzed dental radiographs, and micro-CT images. SR analyzed the histopathology and drafted the manuscript. EC analyzed the histopathology and reviewed the manuscript. NF and SP designed the study, analyzed dental radiographs, and reviewed the manuscript. LF collected samples, analyzed the dental radiographs, and reviewed the manuscript. IP analyzed micro-CT images and reviewed the manuscript.

### Conflict of Interest

The authors declare that the research was conducted in the absence of any commercial or financial relationships that could be construed as a potential conflict of interest.
